# Parcellation of the neonatal cortex using Surface-based Melbourne Children’s Regional Infant Brain atlases (M-CRIB-S)

**DOI:** 10.1038/s41598-020-61326-2

**Published:** 2020-03-09

**Authors:** Chris L. Adamson, Bonnie Alexander, Gareth Ball, Richard Beare, Jeanie L. Y. Cheong, Alicia J. Spittle, Lex W. Doyle, Peter J. Anderson, Marc L. Seal, Deanne K. Thompson

**Affiliations:** 1 Murdoch Children’s Research Institute, Melbourne, Victoria Australia; 2 Department of Medicine, Monash University, Melbourne, Australia; 3 Neonatal services, The Royal Women’s Hospital, Melbourne, Australia; 4 Department of Obstetrics and Gynaecology, The University of Melbourne, Melbourne, Australia; 5 Department of Physiotherapy, The University of Melbourne, Melbourne, Australia; 6 Department of Paediatrics, The University of Melbourne, Melbourne, Australia; 7 Turner Institute for Brain and Mental Health, Monash University, Melbourne, Australia; 8 Florey Department of Neuroscience and Mental Health, The University of Melbourne, Melbourne, Australia

**Keywords:** Paediatric research, Development of the nervous system, Magnetic resonance imaging

## Abstract

Longitudinal studies measuring changes in cortical morphology over time are best facilitated by parcellation schemes compatible across all life stages. The Melbourne Children’s Regional Infant Brain (M-CRIB) and M-CRIB 2.0 atlases provide voxel-based parcellations of the cerebral cortex compatible with the Desikan-Killiany (DK) and the Desikan-Killiany-Tourville (DKT) cortical labelling schemes. This study introduces surface-based versions of the M-CRIB and M-CRIB 2.0 atlases, termed M-CRIB-S(DK) and M-CRIB-S(DKT), with a pipeline for automated parcellation utilizing *FreeSurfer* and developing Human Connectome Project (dHCP) tools. Using *T*_2_-weighted magnetic resonance images of healthy neonates (*n* = 58), we created average spherical templates of cortical curvature and sulcal depth. Manually labelled regions in a subset (*n* = 10) were encoded into the spherical template space to construct M-CRIB-S(DK) and M-CRIB-S(DKT) atlases. Labelling accuracy was assessed using Dice overlap and boundary discrepancy measures with leave-one-out cross-validation. Cross-validated labelling accuracy was high for both atlases (average regional Dice = 0.79–0.83). Worst-case boundary discrepancy instances ranged from 9.96–10.22 mm, which appeared to be driven by variability in anatomy for some cases. The M-CRIB-S atlas data and automatic pipeline allow extraction of neonatal cortical surfaces labelled according to the DK or DKT parcellation schemes.

## Introduction

The delineation of cortical areas on magnetic resonance images (MRI) are considered to be a prerequisite for beginning to understand the complexities of the human brain^1^. Accurate understanding of *development* of the brain is reliant on accurate parcellation of the cerebral cortex, from around the time of normal birth onwards.

*FreeSurfer*^2–5^ is a commonly used cortical extraction and parcellation software suite applicable to *T*_1_-weighted MRI scans of children and adults, and its available parcellation schemes include the Desikan-Killiany (DK)^6^ and Desikan-Killiany-Tourville (DKT)^7^ adult atlases. However, tools tuned for adult brains, such as the adult *T*_1_-based templates and atlases provided in *FreeSurfer*^5,8^, are not directly applicable to neonatal brain images. Tissue signal intensities are different in neonatal brains compared to those in adults^9^. Thus the optimal MRI sequences for the grey and white matter contrast required to identify cortical surfaces differ by age. While *T*_1_-weighting is optimal for adult brains, *T*_2_-weighted contrasts are optimal for neonatal brains. Consequently, specialized algorithms are required in order to contend with neonatal-specific tissue intensities^10,11^. Thus, brain atlases and image segmentation and parcellation tools specific for infants are required.

Many methods for cortical parcellation of infant brain images have focused on warping standardized cortical atlases from adult brains^6,7,12^ onto infant brains^13,14^. However, labelling a neonatal brain image using adult-derived atlases is problematic, due to the inherent difference in anatomy and tissue composition between infant and adult brains^15^. Recently, we introduced the Melbourne Children’s Regional Infant Brain (M-CRIB) atlases^16,17^, which are neonatal-specific, voxel-wise brain atlases. The cortical parcellations were constructed to be compliant with the DK^6^ and DKT^7^ adult cortical parcellation schemes. Each of the M-CRIB atlases are comprised of 10 neonatal brains that have been extensively manually parcellated to accurately reflect brain structures at this time-point. Parcellation of new data has been achieved using multi-atlas label fusion algorithms that probabilistically assign labels to each voxel after warping the set of parcellated atlases to a novel dataset^18,19^.

While accurate labelling can be achieved using voxel-based parcellation schemes^16,18,20^, surface-based registration methods lead to significantly improved alignment of cortical landmarks, including cortical folds, and therefore more accurate placement of areal boundaries^21,22^.

The Developing Human Connectome Project (dHCP) has recently provided a pipeline for segmentation and cortical extraction for *T*_2_-weighted images of neonatal brains^20^, along with an infant cortical surface-based atlas comprising the major lobes^23^. This process segments brain tissue into cerebral and cerebellar grey and white matter, and various subcortical grey matter structures, before extracting an inner and outer cortical surface which is automatically partitioned into lobes. These existing cortical surface extraction tools can be expanded upon by incorporating our M-CRIB brain atlases to provide accurate fine-grained cortical surface-based parcellations, avoiding projection of a non-surface parcellation or a static template. Thus, measures such as cortical thickness, surface area, and curvature for each cortical sub-division of the M-CRIB atlases can subsequently be derived for infant MRI scans.

In this study we aimed to provide neonatal average surface templates and surface-based cortical atlases based on the M-CRIB and M-CRIB 2.0 parcellation schemes, that are compatible with *FreeSurfer* and the dHCP pipelines^2,5,8^. Additionally, we aimed to provide companion scripts to perform cortical surface extraction, surface registration and atlas-based cortical parcellation using novel neonatal *T*_2_*-*weighted brain images. Given the compatibility of the neonatal M-CRIB-S parcellation schemes with the adult DK and DKT schemes, the proposed tools can generate parcellated neonatal cortical regions that are comparable with those obtained using existing tools such as *FreeSurfer* at older time points, enabling longitudinal studies, beginning from the neonatal time-point. This will be valuable for investigating neurological development and disease progression from infancy to adulthood.

## Results

**Figure 1:**
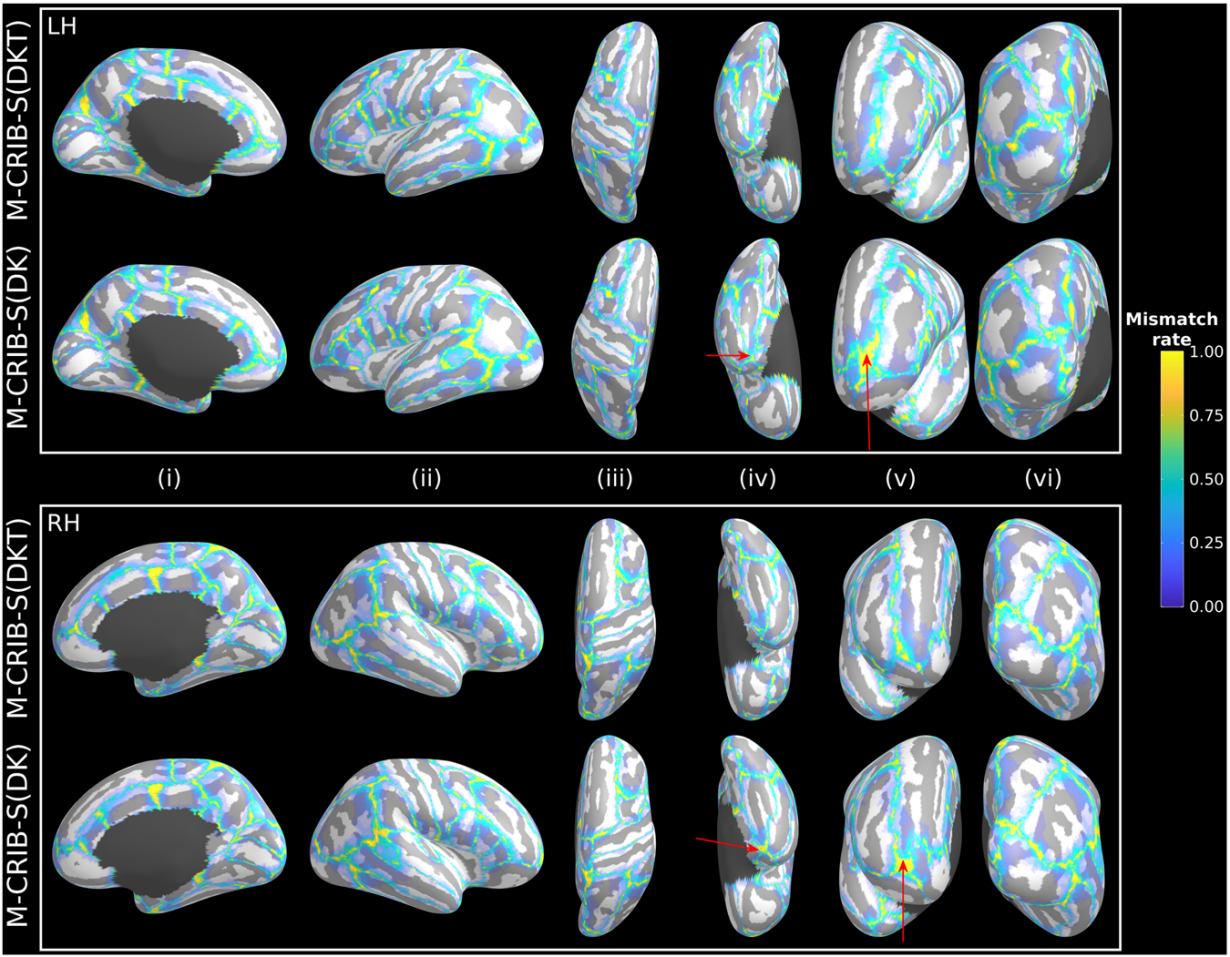
Vertex-wise parcellation mismatch rates for the M-CRIB-S(DKT) and M-CRIB-S(DK) atlases using the Leave-One-Out cross-validation method shown on the template inflated surfaces. Aspects shown are as follows: midline (i), lateral (ii), superior (iii), inferior (iv), frontal (v), and occipital (vi) for the left (LH) and right hemispheres (RH). Warmer colours indicate greater vertex-wise mismatch between automatic and manual labels.

**Figure 2:**
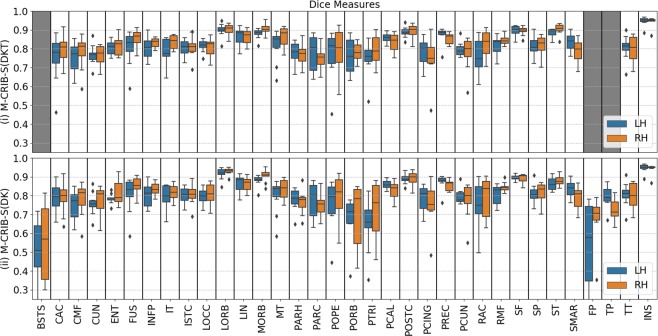
Per-region Dice coefficients for the Leave-One-Out cross-validation test for *labelled* datasets comparing (i) automated with manual M-CRIB-S(DKT) parcellations, and (ii) automated with manual M-CRIB-S(DK) parcellations. The banks of the superior temporal sulcus (BSTS), frontal pole (FP), and temporal pole (TP) regions are greyed out in (i) because they are not present in the DKT parcellation scheme. CAC: Caudal anterior cingulate, CMF: Caudal middle frontal, CUN: Cuneus, ENT: Entorhinal, FUS: Fusiform, INFP: Inferior parietal, INS: Insula, ISTC: Isthmus cingulate, IT: Inferior temporal, LIN: Lingual, LOCC: Lateral occipital, LORB: Lateral orbitofrontal, MORB: Medial orbitofrontal, MT: Middle temporal gyrus, PARH: Parahippocampal, PARC: Paracentral lobule, POPE: Pars opercularis, PORB: Pars orbitalis, PCING: Posterior cingulate, PCAL: Pericalcarine, POSTC: Posterior cingulate, PCUN: Precuneus, PREC: Precentral, PTRI: Pars triangularis, RAC: Rostral anterior cingulate, RMF: Rostral middle frontal, SF: Superior frontal, SMAR: Supramarginal gyrus, SP: Superior parietal, ST: Superior temporal gyrus, TT: Transverse temporal gyrus.

**Figure 3:**
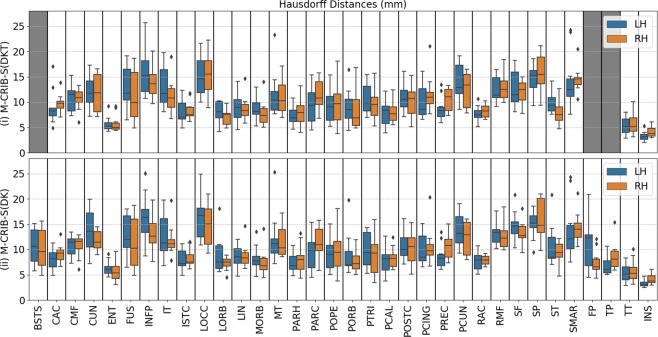
Per-region Hausdorff distances, in mm units, for the Leave-One-Out cross-validation test for *labelled* datasets comparing (i) automated with manual M-CRIB-S(DKT) parcellations and (ii) automated with manual M-CRIB-S(DK) parcellations. The banks of the superior temporal sulcus (BSTS), frontal pole (FP), and temporal pole (TP) regions are greyed out in (i) because they are not present in the DKT parcellation scheme. CAC: Caudal anterior cingulate, CMF: Caudal middle frontal, CUN: Cuneus, ENT: Entorhinal, FUS: Fusiform, INFP: Inferior parietal, INS: Insula, ISTC: Isthmus cingulate, IT: Inferior temporal, LIN: Lingual, LOCC: Lateral occipital, LORB: Lateral orbitofrontal, MORB: Medial orbitofrontal, MT: Middle temporal gyrus, PARH: Parahippocampal, PARC: Paracentral lobule, POPE: Pars opercularis, PORB: Pars orbitalis, PCING: Posterior cingulate, PCAL: Pericalcarine, POSTC: Posterior cingulate, PCUN: Precuneus, PREC: Precentral, PTRI: Pars triangularis, RAC: Rostral anterior cingulate, RMF: Rostral middle frontal, SF: Superior frontal, SMAR: Supramarginal gyrus, SP: Superior parietal, ST: Superior temporal gyrus, TT: Transverse temporal gyrus.

Surface-space versions of the M-CRIB 2.0 and M-CRIB parcellations were derived and named M-CRIB-S(DKT) and M-CRIB-S(DK), respectively. An automated pipeline to segment novel *T*_2_-weighted neonatal images, extract cortical surfaces, and perform cortical parcellation with the M-CRIB-S(DK) and M-CRIB-S(DKT) atlases, was delivered. Labelling accuracy of this pipeline was assessed utilizing a Leave-One-Out atlas generation and automated parcellation strategy for each of the labelled atlas training set (*n* = 10). Results of the Leave-One-Out analysis of labelling accuracy, comparing automated labelling to manually-defined labels in the *labelled* dataset (*n* = 10) are shown in Figs. 1, 2 and 3.

Figure 1 shows the vertex-wise parcellation mismatch rates for both atlases in midline (i), lateral (ii), superior (iii), inferior (iv), frontal (v), and occipital (vi) aspects. Hemisphere-wide vertex-wise agreement rates were similar across parcellation schemes: the average for M-CRIB-S(DKT) was 0.84 [range 0.78–0.87], and the average for M-CRIB-S(DK) was 0.83 [range 0.77–0.87]. The rates of high mismatch are confined to region boundaries, indicating that the bulk of the central portions always agreed with ground truth. Exceptionally high rates of mismatch can be seen for the frontal pole and temporal pole labels in the M-CRIB-S(DK).

Figure 2 displays regional Dice measures for M-CRIB-S(DKT) and M-CRIB-S(DK). Dice scores for both atlases were generally high (0.79–0.83). For the M-CRIB-S(DKT) parcellation scheme, per-region mean Dice measures were similar across hemispheres (left: mean = 0.82, SD = 0.05; right: mean = 0.83, SD = 0.05). In both hemispheres, the highest Dice scores were observed in the insula (left: 0.95; right: 0.94). The lowest Dice score observed in the left hemisphere was for the pars triangularis (0.75), and the lowest Dice score in the right hemisphere was for the posterior cingulate (0.75). For the M-CRIB-S(DK) parcellation scheme, per-region mean Dice measures were similar to those listed above for the M-CRIB-S(DKT) parcellation, and were again similar between hemispheres (left: mean = 0.79, SD = 0.10; right: mean = 0.80, SD = 0.07). The highest Dices scores in each hemisphere were again seen in the insula (left: 0.95; right: 0.94). The lowest Dice scores were outliers observed in the frontal pole in the left hemisphere (0.50), and in the banks of the superior temporal sulcus region in the right hemisphere (0.55).

Figure 3 shows per-region Hausdorff distances, which measure worst-case boundary discrepancy between automatic and manual labels for M-CRIB-S parcellation schemes. For the M-CRIB-S(DKT) parcellation scheme, per-region mean Hausdorff distances were similar between hemispheres (left: mean: 10.22 mm, SD = 2.99 mm; right: mean: 10.13 mm, SD = 2.77 mm). The smallest Hausdorff distances for each hemisphere were both seen in the insula (left: 3.27 mm, right: 4.11 mm). The largest Hausdorff distance for the left hemisphere was observed in the inferior parietal region (16.18 mm), and the largest in the right hemisphere was for the superior parietal region (15.91 mm).

For the M-CRIB-S(DK) parcellation scheme, Hausdorff distances were similar to those for M-CRIB-S(DKT) and were again similar between hemispheres (left: mean = 10.3 mm, SD = 3.08 mm; right: mean = 9.96 mm, SD = 2.67 mm). The smallest Hausdorff distances observed in each hemisphere were again both in the insula (left: 3.23 mm, right: 4.11 mm). The largest Hausdorff distance seen in the left hemisphere was for the inferior parietal region (16.39 mm), and in the right hemisphere was for the superior parietal region (15.96 mm).

**Figure 4:**
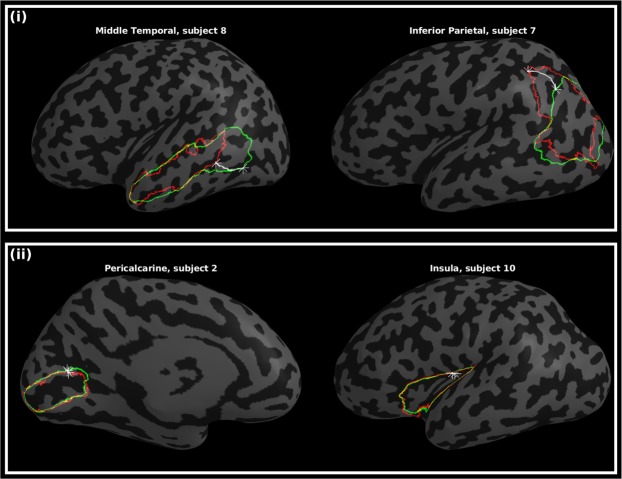
Worst-case (i) and best-case (ii) instances of boundary discrepancies, measured by Hausdorff distances, between estimated (green) and manual (red) label boundaries. The star markers and white paths depict the traversal between nearest neighbours. Other surface vertices are shaded according to curvature, with light and dark grey denoting gyri and sulci, respectively.

Individual measurements of per-subject and per-label Hausdorff distances ranged from 1.9–25.6 mm in M-CRIB-S(DKT) and 2.4–25.3 mm in M-CRIB-S(DK). Figure 4(i) shows some examples of individual worst-case and (ii) best-case Hausdorff distances between ground truth and estimated labels.

**Figure 5:**
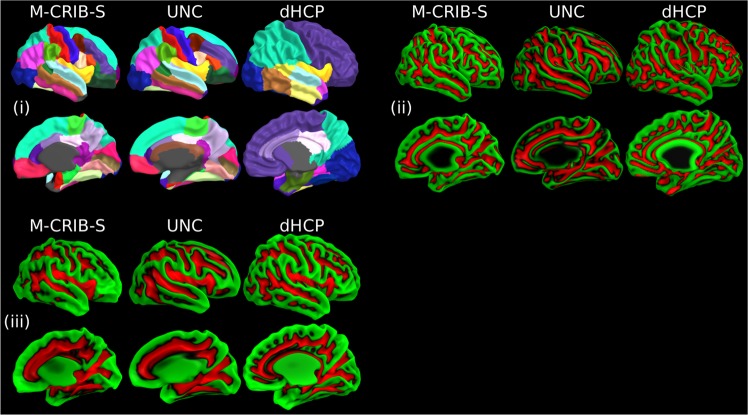
(i) Indicative parcellations, (ii) curvature, and (iii) sulcal depth maps for the M-CRIB-S, University of North Carolina (UNC)^14^, and developing Human Connectome Project (dHCP)^23^ atlases displayed on right hemisphere smoothed white matter surfaces provided for each respective atlas. The M-CRIB-S and UNC parcellations in (i) are based on the DK parcellation scheme. See Bozek *et al*.^23^ for a description of the dHCP parcellation scheme. In (ii) and (iii) vertices are coloured according to whether they reside in a gyrus (green) or a sulcus (red).

Figure 5 shows a comparison of parcellation schemes (i), average curvature (ii) and sulcal depth maps (iii) for the M-CRIB-S, University of North Carolina (UNC)^14^, and dHCP^23^ atlases (right hemisphere smoothed white matter surfaces). The M-CRIB-S parcellation is the result of the automated parcellation method applied to the average surfaces. The UNC and dHCP parcellation labels are those included in the released data packages available for download. The 42-week versions of the UNC and dHCP atlases were chosen as the closest age to the mean of the M-CRIB-S cohort (41.7 weeks postmenstrual age (PMA)).

## Discussion

The primary contribution of this work is the provision of atlases and tools that facilitate cortical surface extraction and parcellation of the neonatal cortex into 31 or 34 regions. Our pipeline is based on *T*_2_-weighted images of neonates around term equivalent age and uses a common adult-compatible parcellation scheme, with neonatal-specific training data. This work extends our previous M-CRIB and M-CRIB 2.0 voxel-based atlases to enable surface-based parcellation of the neonatal cortex.

We have applied this method to a cohort of healthy, term-born infants (mean age at scan = 42.4 weeks). The full age range of subjects suitable for processing under the proposed pipeline will depend on whether tissue intensity contrast is adequate to reliably segment brain structures and extract cortical surfaces, and whether cortical folding complexity is enough to identify all macrostructural morphological features for surface-based template registration and region identification. Tissue segmentation and cortical surface extraction using *DrawEM* and *Deformable* are designed to be compatible with *T*_2_-weighted white/grey matter contrasts visible between 1–5 months^24^ and have been demonstrated on data acquired between 28–45 weeks PMA^20^. From 5 to 8 months’ PMA the *T*_2_-weighted grey/white matter contrast is transitioning to become like that of an adult by about 9 months’ PMA via an isocontrast phase (6–8 months)^24^. As such, tissue segmentation using these protocols would only be expected to work optimally up to approximately 5 months of age, before the *T*_2_-weighted image becomes isointense.

Small-scale cortical folding occurs mostly late in gestation, with many sulci and gyri that define areal boundaries within the M-CRIB-S(DK) and M-CRIB-S(DKT) atlases not being reliably detectable until approximately 40 weeks PMA^13^. It is possible that a sulcus, for example, may not have formed a sufficiently deep groove to be separated from neighbouring gyri. Thus, careful inspection of parcellation results for subjects scanned at ages below 40 weeks’ PMA is required to ensure that the morphological features that define areal boundaries are present. While full-term subjects were chosen for our atlases to avoid confounds introduced by the heterogeneous effects of preterm birth on brain morphology^25^, the lack of this heterogeneity in the training set may limit applicability to preterm or other populations at high risk for brain maldevelopment or malformation. Future work extending our atlases to represent broader ages or clinical groups would be valuable. Nonetheless, the parcellation component may be appropriate for subjects below 40 weeks and beyond 5 months of age, provided cortical surface data were obtained via other preprocessing methods.

Parcellation accuracy of the automated surface-based labelling methods using the M-CRIB-S atlases was quantified using Dice overlap measures and Hausdorff distances using Leave-One-Out cross-validation. Dice overlap scores were high on average and appeared similar in the left and right hemisphere. The per-vertex mismatch rates (Fig. 1) were largely zero for the bulk of the internal portions of most regions. High rates of mismatch for the frontal pole, temporal pole and BSTS labels in the M-CRIB-S(DK) atlas were unsurprising since low labelling accuracy was reported for these regions in the original adult DK atlas^26^, and they were subsequently removed in the adult DKT atlas^27^. The inaccuracy is mainly due to the small size and somewhat arbitrary boundaries of these regions. As such, this is an inherent limitation of the M-CRIB-S(DK) atlas. When compared to accuracy measures presented for the adult DKT atlas, Klein and Tourville^7^ presented Leave-One-Out Dice measures of overlap between *FreeSurfer* parcellations and manual parcellations, which ranged from 0.72–0.98. These values are similar to our Dice overlap results, suggesting that the presented pipeline provides a labelling accuracy consistent with adult parcellation tools. However, it should be noted that the Dice metrics provided in the DKT paper^7^ were not calculated via surface vertices, rather, the parcellations are rasterized into the grey matter ribbon voxels, from which per-label Dice coefficients are computed. While the surface and volumetric Dice measures come from different sources, by construction the labels of the rasterized version correspond to the labels on the surfaces and thus the two are closely related.

Boundary discrepancies between manual and automated labels were measured using Hausdorff distance. The Hausdorff distance is the maximal distance travelled between any two nearest neighbours of manual and automatic label boundary contours. Most regions had Hausdorff distances between 5–8 mm for both M-CRIB-S(DKT) and M-CRIB-S(DK) atlases. Figure 4(i) shows individual instances of worst-case boundary discrepancies in the middle temporal gyrus and inferior parietal labels. The subjects shown, subjects 7 and 8, appeared to exhibit sulcation that varied more than for other subjects in the temporal and parietal cortices and, as a result, the boundaries of these regions were shifted with respect to other training set subjects. An additional confound in manual labelling was that in some instances, label boundaries in the protocols relied on landmarks that were abstract or subject to large individual variability in presence or in morphology. In contrast, the best-case boundary discrepancies (Fig. 4(ii)) feature the insula and pericalcarine regions. The high accuracy of the estimated insula boundary is likely due its particularly well-defined boundaries in the original parcellation protocols, relatively easily identifiable in images and consistent across subjects. Other literature has reported cross-validated boundary discrepancy measures between manual and automated segmentations of the adult DK atlas dataset^6^. Rather than using Hausdorff distances, discrepancies were calculated as average per-vertex distances between manual and automated label boundaries across subjects. Graphical depictions of these average distances appeared to show a maximum discrepancy of 1 mm. Average boundary mismatch is incompatible with the worst-case discrepancy used in this paper and, thus, cannot be directly compared.

The dHCP set of tools are currently available for cortical surface extraction and lobar parcellation. Makropoulos *et al*.^20^ recently highlighted the potential value of incorporating M-CRIB parcellation in these tools^20^, as its compatibility with the DK^6^ adult parcellation may facilitate comparisons of cortical measures between the perinatal and adult time points. Here, we provide a publicly available, surfaced based cortical parcellation that can accomplish this objective.

The value of this surface-based atlas and the associated processing scripts is automated parcellation of the neonatal cortex that is straightforward to employ in longitudinal studies. The processing scripts and the M-CRIB-S(DK) and M-CRIB-S(DKT) atlases were constructed to be used with *FreeSurfer*, to produce compatible output and give a direct correspondence between region-based statistics such as cortical thickness, surface area, and curvature measures at neonatal, childhood and adult timepoints.

## Conclusion

This paper presented the M-CRIB-S(DKT) and M-CBRIB-S(DK) atlases: surface-based versions of the volumetric M-CRIB and M-CRIB 2.0 atlases. It also presented an automated pipeline that involves segmentation of novel *T*_2_-weighted neonatal images, extraction of cortical surfaces, followed by cortical parcellation with the M-CRIB-S(DK) and M-CRIB-S(DKT) atlases, which are neonatal versions of the adult DK and DKT atlases. The curvature template registration targets, average surfaces, labelling training data, and pipeline execution scripts are available. Additionally, for interoperability with the dHCP atlas we have provided a registered version of the spherical template surfaces to be in correspondence to the dHCP template.

## Methods

### Participants

A total of 58 term-born (≥37 weeks’ gestation), healthy neonates (40.2–44.9 weeks post-menstrual age (PMA) at scan, *M* = 42.4, *SD* = 1.2, 26 female) were scanned as control subjects as part of preterm birth studies^28,29^. Criteria for a subject being healthy were no admissions to a neonatal intensive care or special care unit, resuscitation at birth not required, birthweight more than 2.5 kg and no evidence of congenital conditions known to affect development and growth. Ethical approval for the studies was obtained from the Human Research Ethics Committees of the Royal Women’s Hospital and the Royal Children’s Hospital, Melbourne and the research studies complied with the standards of the Declaration of Helsinki. Written informed consent was obtained from parents. Data that exhibited excessive movement or other corrupting artefacts were excluded. This cohort was subdivided into the following two subsets: *labelled* and *unlabelled* subsets. The *labelled* set comprised the ten subjects (40.3–43.0 weeks’ PMA at scan, *M* = 41.7, *SD* = 1.3, 4 female) of the M-CRIB atlas that had been previously selected from this cohort on the basis of minimal motion or other artifact on the *T*_2_-weighted images^16,17^. The *unlabelled* subset consisted of the remaining 48 subjects (40.2–44.9 weeks’ PMA at scan, *M* = 42.6, *SD* = 1.3, 22 female).

### MRI acquisition

All neonate subjects were scanned at the Royal Children’s Hospital, Melbourne, Australia, on a 3 *T* Siemens Magnetom Trio scanner during unsedated sleep. *T*_2_-weighted images were acquired with a turbo spin echo sequence with the following parameters: 1 mm axial slices, flip angle = 120°, repetition time = 8910 ms, echo time = 152 ms, field of view = 192 × 192 mm, in-plane resolution = 1 mm^2^ (zero-filled interpolated to 0.5 × 0.5 × 1 mm in image reconstruction), matrix size = 384 × 384. All *T*_2_-weighted images were resliced to voxel-volume-preserving size of 0.63 × 0.63 × 0.63 mm^16,30^.

### Processing pipeline

**Figure 6:**
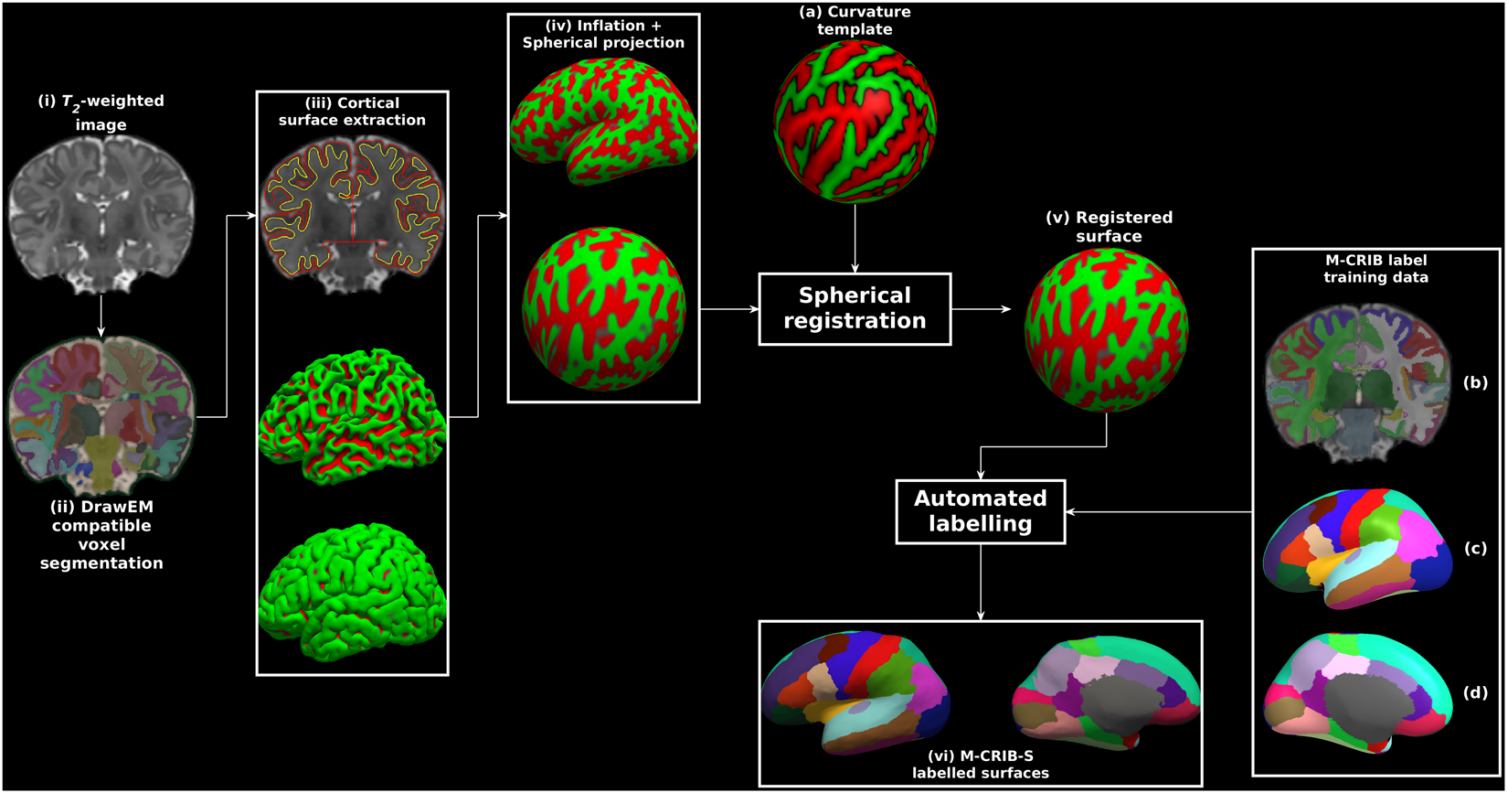
Exemplary surface extraction pipeline output for one labelled subject. Panels show: (i) the original *T*_2_-weighted image; (ii) segmentations according to the *DrawEM* techniques; (iii) *Deformable-*extracted cortical surfaces, where the top panel shows inner (yellow) and outer (red) cortical surfaces overlaid onto the original image, and the middle and bottom panels show lateral aspects of the left hemisphere inner and outer surfaces in 3D; (iv) “inflated” and spherical versions of the white surface; (v) spherical surface registered to the template surface (**a**); (vi) automatic parcellation using the M-CRIB-S(DKT) scheme shown on the subject inflated surface for lateral (left) and medial (right) aspects. The label training data are depicted in volume format (**b**), and in 3D on the average inflated surface for lateral (**c**), and medial (**d**) aspects. Surface vertices in (iii), (iv) and (v) are coloured according to local mean curvature.

The proposed processing pipeline and M-CRIB-S training data is graphically described in Fig. 6.

### Image segmentation

Each image in the *unlabelled* dataset (Fig. 6(i)) was segmented into cerebral white and grey matter (including lobar sub-divisions), cerebellum and various subcortical grey matter structures automatically using the *DrawEM* software package^11,31^. Briefly, this technique non-linearly registered the non-labelled *T*_2_-weighted images to multiple pre-labelled images. The non-labelled image was then segmented using label fusion. The proposed pipeline utilized the wrapper script neonatal-pipeline-v1.1.sh included in *DrawEM* for execution. Figure 6(ii) shows an example voxel-based *DrawEM* segmentation output.

The *labelled* M-CRIB atlas images were already segmented appropriately for *DrawEM* compatibility. Each M-CRIB segmented image comprised manually traced cerebral white and grey matter, cerebellum, basal ganglia and thalamus, cortical, ventricular and other subcortical labels. Tracing protocols for the cortical^16,17^ and subcortical^30^ segmentations have been previously described. Figure 6(b) shows an example M-CRIB-S segmented image.

### Surface extraction

*DrawEM* compatible segmentations containing hemispheric white matter and grey matter, cerebellar, ventricular, brainstem and subcortical grey matter labels were used as input for the *Deformable* module^20,32^ of MIRTK (https://github.com/BioMedIA/MIRTK). *Deformable* used to extract the inner and outer boundaries of each hemisphere of the cerebral cortices for both *labelled* and *unlabelled* datasets. Figure 6(iii) shows inner and outer surfaces overlaid onto the original *T*_2_-weighted image (top), and lateral aspects of the inner (middle) and outer (bottom) surfaces in 3D, respectively.

### Surface inflation and spherical mapping

The proposed pipeline used the *FreeSurfer* tools *mris_inflate* and *mris_sphere*^8^ to construct inflated and spherical versions of the white matter surfaces, respectively. Figure 6(iv) shows exemplary inflated and spherical surface outputs. Default *FreeSurfer* 6.0.0 options were used for both tools with the following exception: the negative triangle removal option “-remove_negative 1” was added to *mris_sphere*. The inflated surfaces exhibited the same gross shape features as those seen when *FreeSurfer* is executed on adult brain images. Specifically, an overall elliptical appearance, a dimple in the vicinity of the insula, and the smooth protrusion of the temporal and occipital poles.

### Curvature template generation

Surface templates, comprised of all *labelled* and *unlabelled* subjects, were constructed using the curvature-based spherical mapping, alignment and averaging method as previously described^2,8^. Briefly, spherical registration involves linear (rotation) and non-linear displacement of vertices in spherical space. The registration algorithm aims to optimise agreement of white and inflated sulcal depth maps of a subject’s surfaces to a template. The use of local curvatures and sulcal depth to drive registration means that corresponding sulci and gyri are aligned. An iterative procedure of aligning spherical surfaces from both the *labelled* and *unlabelled* datasets to the current template, followed by creation of a new template, was performed. The final template curvature and sulcal depth maps were created by averaging all aligned maps (see Fig. 6(a)).

The spherical mapping of each white matter surface onto a common spherical space (Fig. 6(v)) meant that any given point in template space could be mapped to a point on each subject’s white matter surface, and those points were in correspondence across subjects. This enabled average white, pial and inflated surfaces to be constructed using the *FreeSurfer* tool *mris_make_average_surface*, by resampling surfaces onto the 6^th^ order common icosahedron. The 6^th^ order icosahedron was chosen due to having minimal density while still upsampling the original surfaces.

### Surface labelling

For the 10 cases in the labelled dataset, the volumetric M-CRIB and M-CRIB 2.0 labels were projected to the corresponding white matter surface vertices using nearest labelled neighbour projection (See Fig. 6(vi) for parcellation using the M-CRIB-S(DKT) scheme). Label data were individually checked for anatomical accuracy of label placement by one author (B.A.). For both atlases, label placement was considered highly accurate. In a few instances, very minor mislabelling was identified and manually corrected on the relevant surface and corrected volumetrically in some cases for M-CRIB 2.0 data.

**Figure 7:**
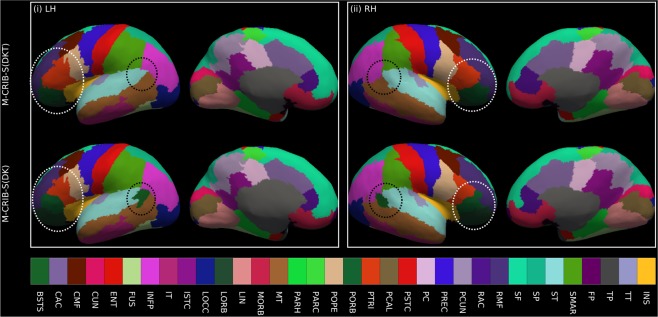
Illustrative surface projections of the manual parcellation for one subject from the *labelled* set using the M-CRIB-S(DKT) and M-CRIB-S(DK) labels for left (LH) and right (RH) hemispheres. The ellipses highlight some differences between M-CRIB-S(DKT) and M-CRIB-S(DK). The white ellipses highlight location disagreements of the lateral orbitofrontal (LORB) and pars orbitalis (PORB) regions between atlases. The black ellipses encompass the banks of the superior temporal sulcus region, which is not present in the DKT. BSTS: Banks of the superior temporal sulcus, CAC: Caudal anterior cingulate, CMF: Caudal middle frontal, CUN: Cuneus, ENT: Entorhinal, FP: Frontal pole, FUS: Fusiform, INFP: Inferior parietal, INS: Insula, ISTC: Isthmus cingulate, IT: Inferior temporal, LIN: Lingual, LOCC: Lateral occipital, LORB: Lateral orbitofrontal, MORB: Medial orbitofrontal, MT: Middle temporal gyrus, PARH: Parahippocampal, PARC: Paracentral lobule, POPE: Pars opercularis, PORB: Pars orbitalis, PCING: Posterior cingulate, PCAL: Pericalcarine, POSTC: Posterior cingulate, PCUN: Precuneus, PREC: Precentral, PTRI: Pars triangularis, RAC: Rostral anterior cingulate, RMF: Rostral middle frontal, SF: Superior frontal, SMAR: Supramarginal gyrus, SP: Superior parietal, ST: Superior temporal gyrus, TP: Temporal pole, TT: Transverse temporal gyrus.

Figure 7 depicts the projection of the M-CRIB and M-CRIB 2.0 labels projected onto the white matter surface generated by *Deformable* for a single *labelled* subject. These surface-space versions of the M-CRIB 2.0 and M-CRIB parcellations are called M-CRIB-S(DKT) and M-CRIB-S(DK), respectively. While similar, the highlighted regions demonstrate some differences including label boundary changes (for example, in lateral orbitofrontal and pars orbitalis) and region removal (banks of the superior temporal sulcus). A comprehensive description of the differences in regions and region boundaries between the M-CRIB and M-CRIB 2.0 parcellations is available in previous publications^16,17^.

### Parcellation training set construction

Parcellation training sets were constructed using the *labelled* set for each M-CRIB-S(DKT) and M-CRIB(DK) cortical label, using the method of Fischl *et al*.^5^. Briefly, for each template, spatial prior distributions for each cortical label were constructed on the surface using the tool *mris_ca_train*. The M-CRIB-S(DKT) parcellation of the average white surface is shown in Fig. 6(c,d).

### Template surface construction

**Figure 8:**
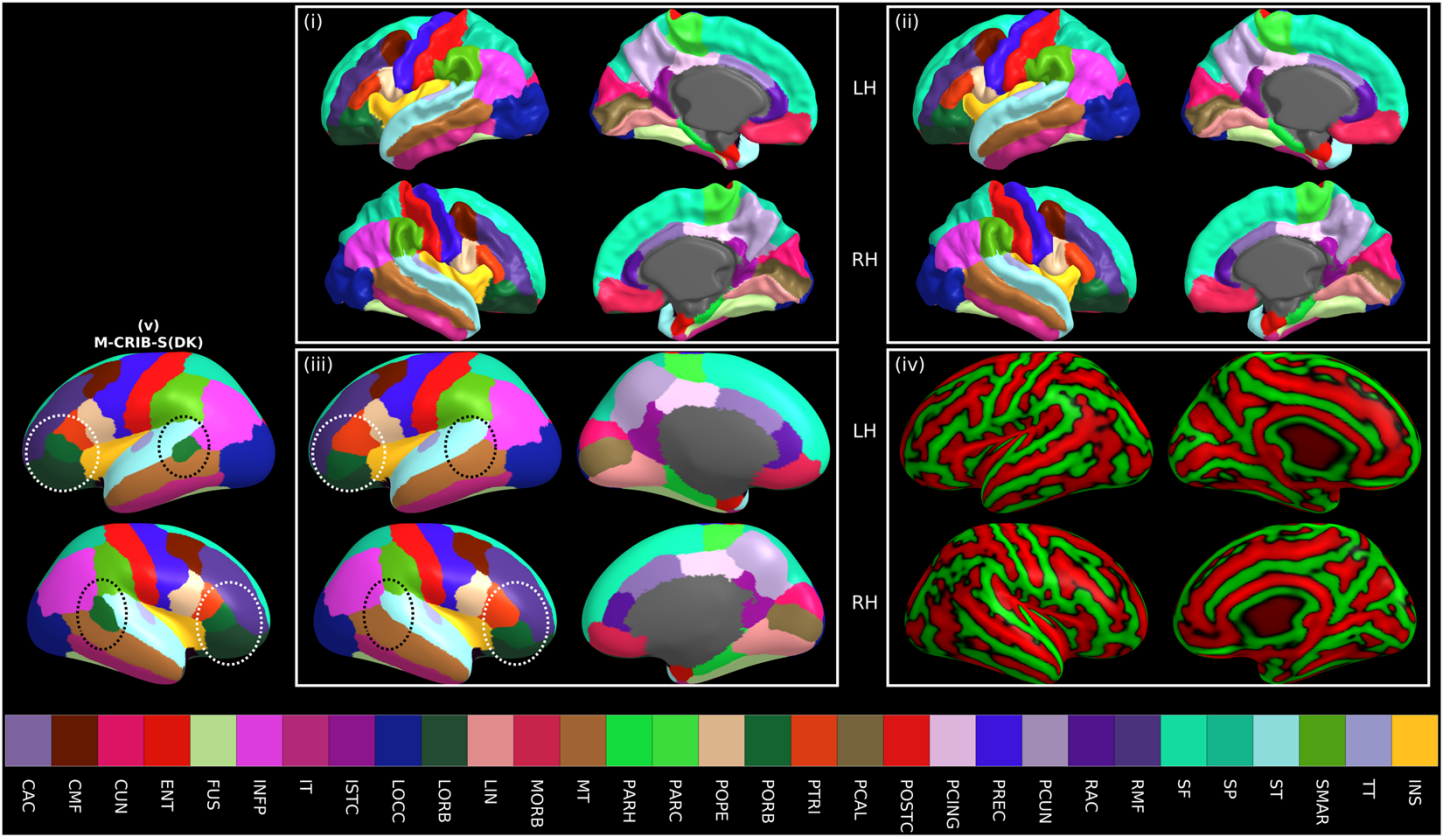
Average white (i), pial (ii), and inflated (iii) left (LH) and right hemisphere (RH) surfaces for all subjects with the vertices coloured according to M-CRIB-S(DKT) labels. The average white matter curvature map is shown on the inflated surfaces in (iv). The lateral view of the M-CRIB-S(DK) atlas is shown in (v). The annotations in panel (iii) and (v) highlight selected differences between the atlases. The white ellipses focus on the lateral orbitofrontal and pars orbitalis regions. The black ellipses centre on the bank of the superior temporal sulcus (BSTS), which is absent in the M-CRIB-S(DKT) atlas. BSTS: Banks of the superior temporal sulcus, CAC: Caudal anterior cingulate, CMF: Caudal middle frontal, CUN: Cuneus, ENT: Entorhinal, FP: Frontal pole, FUS: Fusiform, INFP: Inferior parietal, INS: Insula, ISTC: Isthmus cingulate, IT: Inferior temporal, LIN: Lingual, LOCC: Lateral occipital, LORB: Lateral orbitofrontal, MORB: Medial orbitofrontal, MT: Middle temporal gyrus, PARH: Parahippocampal, PARC: Paracentral lobule, POPE: Pars opercularis, PORB: Pars orbitalis, PCING: Posterior cingulate, PCAL: Pericalcarine, POSTC: Posterior cingulate, PCUN: Precuneus, PREC: Precentral, PTRI: Pars triangularis, RAC: Rostral anterior cingulate, RMF: Rostral middle frontal, SF: Superior frontal, SMAR: Supramarginal gyrus, SP: Superior parietal, ST: Superior temporal gyrus, TP: Temporal pole, TT: Transverse temporal gyrus.

Using both *labelled* and *unlabelled* datasets, we derived group-averaged white (Fig. 8(i)), pial (ii), and inflated surfaces (iii) along with curvature (iv) and sulcal depth maps in a common spherical space. For interoperability with the dHCP and UNC atlases^14,23^, we also provide versions of the M-CRIB-S spherical template surfaces registered to the dHCP 42-week and UNC 42-week spherical template surfaces. M-CRIB-S(DKT) and M-CRIB-S(DK) parcellation maps in each *labelled* subject were transferred to the spherical template and used as the training set for the *FreeSurfer* tool *mris_ca_label*. We applied this labelling to the average white matter surface using the M-CRIB-S(DKT) to illustrate our cortical labelling approach (Fig. 8(i–iii)). For comparison, the M-CRIB-S(DK) labelling is also shown (Fig. 8(v)). These group-average label images may be used for display of statistical analysis results using the M-CRIB-S(DKT) or M-CRIB-S(DK) atlases.

### Parcellation of a novel image

Novel *T*_2_-weighted images can be parcellated using the M-CRIB-S atlas data using the following sequence of processing steps (see Fig. 6): (1) Apply *DrawEM* and *Deformable* to extract white and pial surfaces, (2) Perform surface inflation, spherical projection and registration to the M-CRIB-S surface template using *FreeSurfer* tools, and (3) Use neonatal specific cortical label priors and the automatic labelling tool *mris_ca_label* to parcellate the surfaces. A collection of scripts are provided to execute the pipeline, which can be found along with the M-CRIB-S data on the GitHub page (https://www.github.com/DevelopmentalImagingMCRI/MCRIBS). This pipeline was used to perform cortical parcellation on all *unlabelled* images for validation.

### Parcellation accuracy tests

Parcellation accuracy of the proposed automatic labelling pipeline against manual M-CRIB parcellations was quantified within a Leave-One-Out cross-validation framework. For each of the 10 subjects, curvature templates were constructed using the remaining nine *labelled* subjects and all *unlabelled* subjects. Parcellation training data was constructed from the remaining nine *labelled* subjects. The left-out subject was then segmented and parcellated using the pipeline. Per-region label accuracy was assessed using Dice measures, a metric of overlap, and Hausdorff Distances, a metric of boundary error. The Hausdorff distance between the automatic and manual labelling of a region in one subject is the greatest of all shortest distances between two closed contours. Visualisations of these maximal boundary mismatches are provided.

## Data Availability

The data that support the findings of this study are openly available at https://www.github.com/DevelopmentalImagingMCRI/MCRIBS.

## References

[1] Brett M, Johnsrude IS, Owen AM (2002). The problem of functional localization in the human brain. Nat. Rev. Neurosci..

[2] Fischl B (2012). FreeSurfer. Neuroimage.

[3] Fischl B, Dale AM (2000). Measuring the thickness of the human cerebral cortex from magnetic resonance images. Proc. Natl Acad. Sci..

[4] Fischl B (2002). Whole brain segmentation: automated labeling of neuroanatomical structures in the human brain. Neuron.

[5] Fischl B (2004). Automatically Parcellating the Human Cerebral Cortex. Cereb. Cortex.

[6] Desikan S (2006). An automated labeling system for subdividing the human cerebral cortex on MRI scans into gyral based regions of interest. NeuroImage.

[7] Klein A, Tourville J (2012). 101 Labeled Brain Images and a Consistent Human Cortical Labeling Protocol. Front. Neurosci..

[8] Fischl B, Sereno MI, Tootell RB, Dale AM (1999). High-resolution intersubject averaging and a coordinate system for the cortical surface. Hum. Brain Mapp..

[9] Wang, L. *et al*. Benchmark on Automatic 6-month-old Infant Brain Segmentation Algorithms: The iSeg-2017 Challenge. *IEEE Transactions on Medical Imaging*, 1–1, 10.1109/TMI.2019.2901712 (2019). 10.1109/TMI.2019.2901712 PMC6754324 30835215

[10] Beare RJ (2016). Neonatal Brain Tissue Classification with Morphological Adaptation and Unified Segmentation. Front. Neuroinformatics.

[11] Makropoulos A (2016). Regional growth and atlasing of the developing human brain. Neuroimage.

[12] Tzourio-Mazoyer N (2002). Automated anatomical labeling of activations in SPM using a macroscopic anatomical parcellation of the MNI MRI single-subject brain. Neuroimage.

[13] Shi F (2011). Infant Brain Atlases from Neonates to 1- and 2-Year-Olds. PLoS one.

[14] Wu, Z. *et al*. In *OHBM*.

[15] Richards JE, Sanchez C, Phillips-Meek M, Xie W (2016). A database of age-appropriate average MRI templates. NeuroImage.

[16] Alexander B (2017). A new neonatal cortical and subcortical brain atlas: the Melbourne Children’s Regional Infant Brain (M-CRIB) atlas. Neuroimage.

[17] Alexander B (2019). Desikan-Killiany-Tourville Atlas Compatible Version of M-CRIB Neonatal Parcellated Whole Brain Atlas: The M-CRIB 2.0. Front. Neurosci..

[18] Alexander B (2019). Changes in neonatal regional brain volume associated with preterm birth and perinatal factors. Neuroimage.

[19] Akhondi-Asl A, Warfield SK (2013). Simultaneous truth and performance level estimation through fusion of probabilistic segmentations. IEEE Trans. Med. Imaging.

[20] Makropoulos A (2018). The developing human connectome project: A minimal processing pipeline for neonatal cortical surface reconstruction. Neuroimage.

[21] Ghosh SS (2010). Evaluating the Validity of Volume-Based and Surface-Based Brain Image Registration for Developmental Cognitive Neuroscience Studies in Children 4-to-11 Years of Age. NeuroImage.

[22] Coalson TS, Van Essen DC, Glasser MF (2018). The impact of traditional neuroimaging methods on the spatial localization of cortical areas. Proc. Natl Acad. Sci..

[23] Bozek J (2018). Construction of a neonatal cortical surface atlas using Multimodal Surface Matching in the Developing Human Connectome Project. Neuroimage.

[24] Wang L (2012). 4D Multi-Modality Tissue Segmentation of Serial Infant Images. PLoS one.

[25] Volpe JJ (2009). Brain injury in premature infants: a complex amalgam of destructive and developmental disturbances. Lancet Neurol..

[26] Desikan RS (2006). An automated labeling system for subdividing the human cerebral cortex on MRI scans into gyral based regions of interest. Neuroimage.

[27] Klein A, Tourville J (2012). 101 labeled brain images and a consistent human cortical labeling protocol. Front. Neurosci..

[28] Spittle AJ (2014). Neurobehaviour between birth and 40 weeks’ gestation in infants born <30 weeks’ gestation and parental psychological wellbeing: predictors of brain development and child outcomes. BMC pediatrics.

[29] Walsh JM, Doyle LW, Anderson PJ, Lee KJ, Cheong JL (2014). Moderate and late preterm birth: effect on brain size and maturation at term-equivalent age. Radiology.

[30] Loh WY (2016). A New MRI-Based Pediatric Subcortical Segmentation Technique (PSST). Neuroinformatics.

[31] Makropoulos A (2014). Automatic whole brain MRI segmentation of the developing neonatal brain. IEEE Trans. Med. Imaging.

[32] Schuh, A. *et al*. In 2017 *IEEE 14th International Symposium on Biomedical Imaging (ISBI 2017*). 800–803.

